# Evaluating the risk factors for bone cement implantation syndrome in hip surgeries - A cross sectional observational study

**DOI:** 10.6026/973206300213994

**Published:** 2025-11-15

**Authors:** Rashmi Pal, J. Bindu Niharika, Pooja Vaskle

**Affiliations:** 1 Department of Anesthesiology, MGM Medical College and M.Y. Hospital, Madhya Pradesh, India

**Keywords:** Bone cements implantation syndrome (BCIS), intraoperative complications, risk factors, hemodynamic changes, anesthetic management

## Abstract

Bone cement implantation syndrome (BCIS) is a serious complication seen in cemented hip surgeries, causing hypoxia, hypotension and cardiovascular collapse but Indian studies on risk factors are limited. In this prospective study of 50 patients at MGM Medical College Indore, BCIS occurred in 20% cases (n=10), mostly mild Grade 1 (80%). Elderly patients (mean age 77.7 ± 4.6 years, p=0.001), ASA grade 3 (p=0.001), COPD (40%, p=0.001), hypertension (70%, p=0.012) and long-stem prosthesis (60%, p=0.006) showed higher risk. BCIS patients had significant fall in heart rate, BP and oxygen levels within first 30 minutes of cement insertion, needing urgent vasopressors and steroids. Gender, diabetes and fracture type were not linked to BCIS, highlighting the need for early identification and close monitoring in high-risk groups.

## Background:

Hip fractures are a growing concern worldwide, especially among elderly patients, and are projected to exceed six million cases by 2050 because of ageing and osteoporosis [1]. Cemented hip arthroplasty using polymethyl methacrylate (PMMA) is commonly preferred in such patients to improve fixation and reduce implant loosening [2]. During cemented arthroplasty, PMMA seals the femoral canal, raising intramedullary pressure that drives marrow emboli into circulation, causing haemodynamic instability and leading to Bone Cement Implantation Syndrome (BCIS) with hypoxia, hypotension, and possible cardiac arrest [3]. Donaldson *et al.* defined the clinical grades of BCIS-Grade 1 (mild), Grade 2 (severe with unconsciousness), and Grade 3 (cardiovascular collapse)-and described early signs such as a sudden fall in end-tidal CO_2_ under general anaesthesia or dyspnoea and confusion under regional block [4]. Older age, higher ASA grade, COPD, hypertension, and osteoporosis have been recognised as important risk factors [5]. Management focuses on oxygenation, vasopressors, inotropes, corticosteroids, and antihistamines [6]. Olsen *et al.* reported BCIS incidence of 28 percent in cemented hip surgeries with mortality above 90 percent in severe cases [7]. Therefore, it is of interest to evaluate the risk factors for bone cement implantation syndrome in hip surgeries.

## Materials and Methods:

Following approval from the Institutional Ethics Committee[IEC/MGM/Sept23/101] this cross-sectional observational study was conducted in the Department of Anaesthesiology, MGM Medical College and M.Y. Hospital, Indore, Madhya Pradesh, India, over a period of 12 months (October 2023 to October 2024). A total of 50 patients scheduled for elective cemented hip arthroplasty in the Department of Orthopaedics were recruited after meeting the defined inclusion criteria. All participants were explained the purpose, protocol and procedures of the study in their vernacular language. Written informed consent was obtained from each participant before enrolment in accordance with ethical guidelines and strict confidentiality of patient data was maintained throughout the study. The sample size was calculated using Cochran's formula based on the findings of a prior study reporting a 3.02% incidence of Bone Cement Implantation Syndrome (BCIS) [14], with a 95% confidence interval (Z = 1.96) and a 5% margin of error, resulting in a minimum required sample of 50 participants. Inclusion criteria comprised patients of either sex, aged above 20 years, with an ASA physical status of Grade II to IV, undergoing cemented hip arthroplasty for hip fractures. Patients who declined to participate were excluded from the study. Written informed consent was obtained from all participants in their vernacular language after the study protocol was explained in detail.

## Methodology:

## Preoperative assessment and preparation:

A detailed pre-anaesthetic evaluation was performed for each patient. Baseline investigations included complete blood count (CBC), random blood sugar (RBS), renal function tests (RFT), chest X-ray and electrocardiography (ECG). Echocardiography was done in cases with abnormal ECG findings. ASA grading was assigned to each patient. Patients were instructed to remain nil per oral for 8 hours before surgery.

## Intraoperative monitoring and anaesthesia:

Upon arrival in the operating theatre, intravenous access was established using an 18G cannula. Patients were preloaded with Ringer's lactate at 5 ml/kg/hr. Standard intraoperative monitoring was instituted, including electrocardiography (ECG), pulse oximetry (SpO_2_) and non-invasive blood pressure (NIBP). Oxygen was administered via nasal prongs at 2 L/min. Subarachnoid block was performed using 0.5% heavy bupivacaine, administered via a 25G or 26G spinal needle under aseptic precautions, in sitting or lateral decubitus position. Baseline vital parameters were recorded before administration of anaesthesia and then at 5-minute intervals during the first 30 minutes of surgery and every 30 minutes thereafter until the end of the procedure.

## Assessment of BCIS:

The presence and severity of BCIS were evaluated using Donaldson's classification [2], which defines three grades based on intraoperative hypoxia, hypotension, or cardiovascular collapse. Perioperative measures taken to prevent or manage BCIS-such as administration of corticosteroids, antihistamines, vasopressors, or inotropes-were recorded. Intraoperative blood loss, duration of surgery and type of prosthesis used were also noted.

## Statistical analysis:

Data was systematically collected using a structured proforma. Entries were made in Microsoft Excel and analyzed using SPSS software version 25.0. Continuous variables were expressed as mean ± standard deviation (SD), while categorical data were presented as frequencies and percentages. After assessing for normality, comparisons between continuous variables were made using the unpaired t-test and comparisons between proportions were done using the Z-test for two-sample proportions. A p-value <0.05 was considered statistically significant. Final results were presented in tabular and graphical formats as appropriate.

## Results:

In the present study involving 50 patients undergoing cemented hip arthroplasty, bone cement implantation syndrome (BCIS) was observed in 10 patients (20%). Grade 1 BCIS was most common (80%), while Grades 2 and 3 were seen in 10% each. BCIS occurred exclusively in patients aged >60 years, with 70% between 61-80 years and 30% above 80 years. Gender distribution showed no significant association with BCIS, with equal numbers of males and females affected in the BCIS group and a mild male predominance in the non-BCIS group (p = 0.568). A significant association was found between ASA physical status and BCIS occurrence. While all non-BCIS patients were ASA Grade 2, 40% of BCIS cases were ASA Grade 3 (p = 0.001), indicating that poorer preoperative physical status increases BCIS risk. Comorbidity analysis further revealed significantly higher prevalence of chronic obstructive pulmonary disease (COPD), hypertension, osteoporosis and use of long-stem prostheses in the BCIS group. COPD was present in 40% of BCIS patients and none in the non-BCIS group (p = 0.001). Hypertension was more frequent in BCIS patients (70%) compared to those without BCIS (27.5%, p = 0.012), as was osteoporosis (60% vs. 5%, p = 0.001). Long-stem prosthesis usage was also significantly higher in BCIS patients (60%) compared to the non-BCIS group (17.5%, p = 0.006) (Table 1 - see PDF). The mean age in the BCIS group (77.7 ±4.57 years) was significantly higher than in the non-BCIS group (49.45 ±15.35 years), indicating age as a strong risk factor (p = 0.001). The mean operative time was slightly shorter in the BCIS group (112.5 ±31.02 min) compared to the non-BCIS group (115.8 ±37.76 min), but not significant (p = 0.800). Similarly, the mean intraoperative blood loss was lower in BCIS patients (71.00 ±57.05 ml) than in those without BCIS (95.88 ±163.98 ml), though this difference was not statistically significant (p = 0.641) (Table 2 - see PDF). These findings collectively underscore that increasing age, poor ASA status and specific comorbid conditions such as COPD, hypertension and osteoporosis, along with the use of long-stem implants, are significant predictors of BCIS in cemented hip arthroplasty.

In the present study, intraoperative monitoring revealed significant hemodynamic differences between patients who developed Bone Cement Implantation Syndrome (BCIS) and those who did not. While baseline values for heart rate (HR), systolic blood pressure (SBP), diastolic blood pressure (DBP) and oxygen saturation (SpO_2_) were comparable at induction, notable deviations occurred following cement insertion. BCIS patients had exhibited significant bradycardia post-cementation. At 5 minutes, the HR dropped to 70 ±5.14 bpm compared to 77.38 ±9.45 bpm in non-BCIS patients (p = 0.022), with a further decline at 10 and 15 minutes (p = 0.003) (Figure 1 - see PDF), before normalizing after 30 minutes. SBP also decreased significantly in BCIS cases, dropping to 109.2 ±16.5 mmHg at 10 minutes (p = 0.009) and reaching a nadir of 92 ±12.42 mmHg at 20 minutes (p < 0.001). Differences persisted up to 120 minutes (Figure 2 - see PDF) but resolved by the end of surgery. DBP followed a similar pattern (Figure 3 - see PDF), with the lowest values also at 20 minutes (56.6 ±7.99 mmHg, p < 0.001). SpO_2_ levels declined significantly during the first 30 minutes, with the lowest at 20 minutes (97.2 ±2.86% vs. 99.2 ±0.69%, p < 0.001), but normalized thereafter (Figure 4 - see PDF).

These findings highlight the transient yet critical nature of BCIS-related intraoperative instability. Notably, 100% of patients without BCIS required no intervention, whereas all BCIS patients required some form of pharmacologic support, most commonly Phenylephrine with Hydrocortisone (Figure 5 - see PDF).

## Discussion:

This cross-sectional observational study was conducted in the Department of Anaesthesiology at MGM Medical College and M.Y. Hospital, Indore, involving 50 patients scheduled for elective cemented hip arthroplasty. The primary objective was to evaluate the perioperative risk factors and hemodynamic alterations associated with Bone Cement Implantation Syndrome (BCIS). Out of the total participants, 10 patients (20%) developed BCIS, with the majority classified as Grade 1 (80%) and Grades 2 and 3 constituting 10% each, based on the criteria propose by Donaldson *et al.* [2]. Age was found to be a significant risk factor for BCIS. Patients who developed BCIS had a significantly higher mean age (77.7 ±4.57 years) compared to non-BCIS patients (49.45 ±15.35 years), with a highly significant p-value of 0.001. Notably, all BCIS cases occurred in patients older than 60 years. These observations are consistent with earlier studies. Rassir Rachid *et al.* (2021) identified advanced age (above 75 years) as a key determinant of BCIS severity (OR 1.57) due to diminished cardiopulmonary reserve and greater comorbidity burden [8]. Similarly Weingartner *et al.* reported a mean BCIS patient age of 81.1 ±10.0 years while Bhadani *et al.* (2024) found BCIS predominantly in individuals over 60 years with multiple comorbidities [9, 10]. Gender distribution was not statistically associated with BCIS (p = 0.567). Equal numbers of males and females were affected in the BCIS group, while males slightly predominated in the non-BCIS group. Yang *et al.* also reported no significant sex-based differences in BCIS incidence [11]. Also studies by Ming-Che Tsai *et al.* and Jain *et al.* observed a higher incidence in males, attributing this to increased intramedullary pressures and a higher prevalence of cardiovascular comorbidities among men [12, 15]. ASA physical status showed a strong correlation with BCIS. All non-BCIS patients were ASA Grade 2, while 40% of BCIS patients were ASA Grade 3 (p = 0.001). This finding is supported by Olsen *et al.* (2014) who linked higher ASA grades with increased postoperative mortality in cemented hip arthroplasty patients and by Rassir *et al.* who identified ASA Grades III-IV as independent predictors of BCIS (OR 1.58) [7, 8]. Comorbid conditions such as hypertension, COPD and osteoporosis were significantly associated with BCIS occurrence. Hypertension was found in 70% of BCIS patients versus 27.5% in the non-BCIS group (p = 0.012). This aligns with Olsen *et al.* who observed increased BCIS severity in hypertensive patients, particularly those using beta-blockers or ACE inhibitors, which may impair hemodynamic compensation [7]. Bhadani *et al.* also noted hypertension as a contributing factor due to reduced baroreflex sensitivity and vascular stiffness [10]. COPD was present in 40% of BCIS patients but was absent among non-BCIS individuals (p = 0.001), reinforcing previous findings by Olsen *et al.* and Bhadani *et al.* who emphasized the role of impaired gas exchange in increasing susceptibility to BCIS-related hypoxia and cardiovascular compromise [7, 10]. Osteoporosis was also significantly more common among BCIS patients (60%) than in those without BCIS (5%) (p = 0.001). This is in line with Chulsomlee *et al.* who found a high prevalence of BCIS among osteoporotic fracture patients and with Bhadani *et al.* who attributed the risk to increased marrow and fat embolization during cement pressurization in osteoporotic bone [10, 13]. The use of long-stem prostheses was significantly more frequent in BCIS cases (60%) compared to non-BCIS cases (17.5%) (p = 0.006). Herrenbruck *et al.* reported that long-stem implants in un-instrumented canals generate high intramedullary pressures during cementation, resulting in greater embolic load and BCIS risk [14].

Hemodynamically BCIS patients experienced significantly lower heart rates at 5, 10, 15 and 20 minutes post-cementation (p < 0.05), normalizing by 25 minutes. This mirrors findings by Soleimanha *et al.* who observed transient bradycardia after cement application and Park *et al.* who reported reduced cardiac output in elderly BCIS patients, often requiring higher vasopressor support [15, 16]. Systolic blood pressure in BCIS patients was significantly lower from 10 to 120 minutes post-implantation (p < 0.05), while diastolic pressure showed significant drops between 10-30 and 90 minutes, indicating transient but critical cardiovascular compromise. Similar trends were documented by Soleimanha *et al.*, Yang *et al.* and Bhadani *et al.* [10, 11 and 15]. Oxygen saturation (SpO_2_) also declined significantly between 10 and 30 minutes post-cementation in BCIS patients, returning to normal thereafter. This transient hypoxia corresponds with embolic obstruction in the pulmonary vasculature, as described by Rassir *et al.*, Bonfait *et al.* and Bhadani *et al.* [8, 10 and 17]. Of the 10 BCIS cases, all required pharmacological intervention. Phenylephrine with hydrocortisone was used in 12% of cases, ephedrine alone in 6% and a combination of hydrocortisone and ephedrine in 2%. These medications addressed vasodilation and inflammation. Park *et al.* similarly emphasized increased vasopressor requirements in elderly BCIS patients [16]. Also a study reported a high BCIS incidence (74%) in cancer patients undergoing cemented hip arthroplasty, identifying advanced age and cardiopulmonary disease as major predictor which supports our findings that age, ASA grade, and poor cardiopulmonary reserve remain the most consistent risk factors across populations [18]. The limitations of this study include its small sample size, single-center scope, lack of long-term follow-up and the absence of advanced hemodynamic monitoring. These constraints underscore the need for larger, multicentric Indian studies to better understand and manage BCIS in diverse patient populations.

## Conclusion:

BCIS as a significant perioperative complication in cemented hip arthroplasty, with strong associations to advanced age, higher ASA grade, hypertension, COPD, osteoporosis and long-stem prostheses. Gender showed no correlation. BCIS was marked by transient hemodynamic instability post-cementation. Thus, we show the need for preoperative risk assessment, optimization of comorbidities and vigilant intraoperative monitoring, emphasizing the anaesthesiologist's critical role in improving patient outcomes.
